# Supramolecular polymer-based transformable material for reversible PEGylation of protein drugs

**DOI:** 10.1016/j.mtbio.2021.100160

**Published:** 2021-11-16

**Authors:** Kosei Utatsu, Tetsuya Kogo, Toru Taharabaru, Risako Onodera, Keiichi Motoyama, Taishi Higashi

**Affiliations:** a Graduate School of Pharmaceutical Sciences, Kumamoto University, 5-1 Oe-honmachi, Chuo-ku, Kumamoto, 862-0973, Japan; b Priority Organization for Innovation and Excellence, Kumamoto University, 5-1 Oe-honmachi, Chuo-ku, Kumamoto, 862-0973, Japan

**Keywords:** Polyrotaxane, Polyethylene glycol, Supramolecular material, Protein, Pharmaceutical property

## Abstract

We herein developed a transformable mixing-type material for reversible PEGylation of protein drugs using a supramolecular backbone polymer, that is, polyrotaxane possessing both amino groups and PEG chains (PEG–NH_2_–PRX). We expected that PEG–NH_2_–PRX provides amino groups to interact with protein drugs on demand because the mobility of amino groups in PEG–NH_2_–PRX was high. In fact, PEG–NH_2_–PRX formed complexes with protein drugs efficiently compared to PEGylated amino-dextran (PEG–NH_2_–DEX), a control material fabricated with a macromolecular backbone polymer. Moreover, PEG–NH_2_–PRX markedly improved the stability of antibodies and prolonged the hypoglycemic effects of insulin without loss of bioactivity, compared to PEG–NH_2_–DEX. These findings suggest that the supramolecular material, PEG–NH_2_–PRX, is a promising reversible PEGylation material for protein drugs compared to macromolecular materials.

## Graphical abstract

**Figure:**
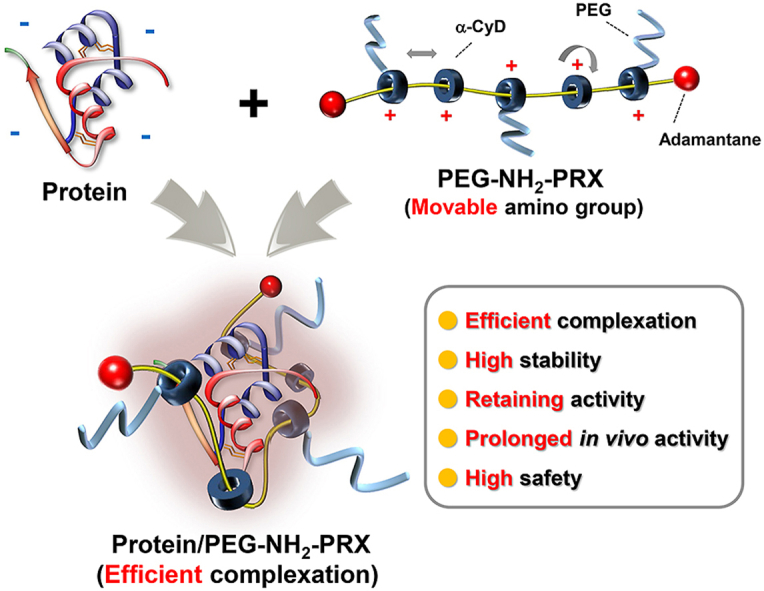

## 1 Introduction

Recent years have seen extensive development of protein drugs, with worldwide sales in 2020 showing that protein drugs constitute five of the top ten pharmaceuticals used [1]. However, protein drugs often show low physicochemical stability and short blood retention time [2]. Thus, pharmaceutical additives such as sugars, amino acids, and polymers are used to increase the stability of these formulations and make them suitable for use. However, these additives do not generally have any considerable effect on the pharmaceutical properties of proteins [3]. Therefore, bioconjugation methods such as polyethylene glycol (PEG)-modification are often used [3]. However, covalent PEGylation dramatically reduces the bioactivity of protein drugs; for example, interferon-α 2a and insulin lose 93% and 94% of their activity by PEGylation, respectively [4,5]. Moreover, the synthesis and purification of PEGylated proteins are laborious processes, and proteins are often lost during these steps, leading to high costs.

Recently, mixing-type PEGylation materials have attracted considerable attention because of their reversible properties and convenient processing, and their monovalent and multivalent PEGylation types have been reported (Fig. S1a–c) [[6], [7], [8], [9]]. Multivalent types are further classified into block type- and grafted type-PEGylation (Fig. S1b, c). For instance, Asayama et al. developed cholesterol-appended PEG (monovalent type), which allowed reversible PEGylation of insulin through hydrophobic interaction with the cholesterol moiety [6]. Appel's group reported PEGylated cucurbit [7]uril for monovalent PEGylation of insulin through a host-guest interaction between N-terminal aromatic residue of insulin and cucurbit [7]uril [7,8]. Tsiourvas et al. fabricated multivalent PEGylation materials for insulin using oligolysin- or oligoarginine-appended PEG [9]. Further, we have previously reported a monovalent-type PEGylation method using host-guest interactions between cyclodextrin (CyD) and adamantane [[10], [11], [12]]. However, the interactions between these materials and proteins are generally weak, leading to facile dissociation *in vivo* and subsequent adverse effects on the prolonged blood retention of proteins. This is probably because of few interaction points for monovalent types and steric hindrance of PEG chains against the interaction points for multivalent types. Therefore, development of new PEGylation materials that can achieve efficient multivalent interaction with protein drugs while avoiding steric hindrance of PEG chains is required.

Polyrotaxanes (PRXs) are mechanically interlocked supermolecules obtained by threading linear compounds through a number of macrocyclic compounds and capping their terminals with bulky compounds. PRXs have attracted considerable attention owing to their unique properties and have been widely used as topological materials [13]. Moreover, CyD-based PRXs [14] have been widely used to fabricate biomaterials because of their facile preparation, high yield, safety profile, and low cost [[15], [16], [17]]. Importantly, the CyD molecules in PRX can be moved onto an axile molecule [18]. Therefore, ligands such as sugars and amino acids modified on CyD molecules in PRX can be easily moved, which then interact strongly with target molecules, such as lectin and transporters [[19], [20], [21], [22]]. Tamura et al. reported that cationic PRX efficiently forms a complex with anionic β-galactosidase, and is useful for its intracellular delivery [23,24]. Thus, the mobility of functionalized PRXs allows for efficient complex formation with proteins; therefore, PRXs could be useful as backbone polymers for fabricating mixing-type PEGylation materials.

With this background, in the present study, we initially prepared a PRX-based supermolecule possessing amino groups (NH_2_-PRX) that efficiently interacted with acidic proteins. Then, transformable mixing-type PEGylation materials were prepared by partially grafting PEG chains with NH_2_-PRX (PEG–NH_2_–PRX). We hypothesized that the amino groups in PEG–NH_2_–PRX would interact with acidic protein drugs while avoiding the steric hindrance of PEG chains, resulting in efficient complexation and indirect PEGylation without the formation of covalent bonds (Fig. S1d). Samples with low mobility of amino groups, amino-dextran (NH_2_-DEX), and PEGylated NH_2_-DEX (PEG–NH_2_–DEX) were prepared as controls. In this study, insulin (isoelectric point; i.p. 5.3), hyaluronidase (i.p. 5.7), immunoglobulin G (IgG) (i.p. 6∼), and lysozyme (i.p. 11, a negative control) were used as model proteins, and the effects of PEG–NH_2_–PRX on their stability and duration of bioactivity were evaluated.

## 2 Materials and methods

### 2.1 Materials

Human insulin, hen egg lysozyme, bovine hyaluronidase, human IgG, and panitumumab (Vectibix®) were obtained from Fujifilm Wako Pure Chemical Corporation (Osaka, Japan), Sigma Chemicals (St. Louis, MO, USA), MP Biomedicals (Irvine, CA), Equitech-Bio (Kerrville, TX), and Takeda Pharmaceutical Company, Ltd. (Osaka, Japan), respectively. α-CyD was supplied by Nihon Shokuhin Kako (Tokyo, Japan). PEG (MW 20 ​kDa) was purchased from Fujifilm Wako Pure Chemical Corporation (Osaka, Japan). Sunbright® ME-020 CS (mPEG-NHS) was obtained from NOF (Tokyo, Japan). All other chemicals and solvents were of analytical reagent grade, and deionized double-distilled water was used throughout the study.

### 2.2 Preparation of PRX

PRX was prepared according to the method described by Araki [25]. PEG (MW 20 ​kDa) (50 ​g) and carbonyldiimidazole (CDI) (1.0 ​g) were dissolved in tetrahydrofuran (THF) (200 ​mL) and stirred under an N_2_ atmosphere for 18 ​h at 50 ​°C. The reactant was added dropwise to ethylenediamine (3 ​mL) and stirred for 2 ​h at 50 ​°C. After adding ethanol (200 ​mL) and standing for 2 ​h at −20 ​°C, the precipitates were collected by centrifugation and dried under reduced pressure.

To obtain polypseudorotaxane (PpRX), PEG-bis amine (3.0 ​g) was added to a 12% (w/v) α-CyD aqueous solution (100 ​mL). After stirring overnight at 4 ​°C, the precipitates were collected by centrifugation and dried by lyophilization.

To obtain PRX, 1-adamantaneacetic acid (2.45 ​g), BOP reagent (5.25 ​g), 1-hydroxybenzotriazole (HOBt) (1.75 ​g), and *N*-ethyldiisopropylamine (2.28 ​mL) were dissolved in dimethylformamide (DMF) (100 ​mL), and PpRX (14.0 ​g) was added. After stirring for 48 ​h at 4 ​°C under an N_2_ atmosphere, the precipitates were collected by centrifugation and washed with methanol/DMF (1:1 v/v) and with methanol two times, respectively. The resulting product was dissolved in dimethyl sulfoxide (DMSO) and precipitated in an excess of water. The above procedure was repeated 3 times, and the obtained precipitates were dried by lyophilization.

### 2.3 Preparation of NH_2_-PRX

NH_2_-PRX was prepared according to the method reported previously [26]. PRX (8.41 ​g) and CDI (8.29 ​g) were dissolved in DMSO (250 ​mL) and stirred overnight at room temperature under an N_2_ atmosphere. The reactant was added dropwise to 1,2-bis(2-aminoethoxy)ethane (75.8 ​mL) and stirred overnight at room temperature under an N_2_ atmosphere. After dialysis (Spectra/Por® membrane MWCO: 10 ​kDa) against water, the sample was dried by lyophilization.

### 2.4 Preparation of NH_2_-DEX

NH_2_-DEX was prepared according to the method reported previously [26]. Dextran (MW 70 ​kDa) (7.56 ​g) and CDI (10.07 ​g) were dissolved in DMSO (250 ​mL), and stirred overnight at room temperature under an N_2_ atmosphere. The reactant was added dropwise to 1,2-bis(2-aminoethoxy)ethane (92.1 ​mL) and stirred overnight at room temperature under an N_2_ atmosphere. After dialysis (Spectra/Por® membrane MWCO: 10 ​kDa) against water, the sample was dried by lyophilization.

### 2.5 Preparation of PEG–NH_2_–PRX

NH_2_-PRX (134.1 ​mg) and mPEG-NHS (250 ​mg) were dissolved in DMSO (12.5 ​mL) and stirred for 24 ​h at room temperature. The reactant was dialyzed (Spectra/Por® membrane MWCO: 50 ​kDa) against water and dried by lyophilization.

### 2.6 Preparation of PEG–NH_2_–DEX

NH_2_-DEX (194.3 ​mg) and mPEG-NHS (192.3 ​mg) were dissolved in DMSO (100 ​mL) and stirred for 24 ​h at room temperature. The reactant was dialyzed (Spectra/Por® membrane MWCO: 50 ​kDa) against water and dried by lyophilization.

### 2.7 Structural characterization

The ζ-potential values were determined by dynamic light scattering using a Zetasizer Nano ZS apparatus (Malvern Instruments, Worcestershire, UK). ^1^H NMR spectra were recorded at 25 ​°C on a Jeol JNM-ECP500 spectrometer (Tokyo, Japan) operating at 500 ​MHz. The solid samples were dissolved in 0.6 ​mL of deuterated DMSO (DMSO-*d*_*6*_) or deuterium oxide (D_2_O). Circular dichroism (CD) spectra were measured using a J-820 dichroism spectrometer (JASCO, Tokyo, Japan) at 25 ​°C. The flow rate of nitrogen gas was 3 ​L/min. The time constant was 4 ​s. The scanning speed was 50 ​nm/min. Molecular ellipticity was expressed as the average molecular ellipticity per amino acid residue equivalent.

### 2.8 Interaction assay by gel electrophoresis

Insulin (0.58 ​mg/mL) and NH_2_-PRX (2.0 ​mg/mL) were dissolved in HBSS buffer. The solution (40 ​μL) was mixed with 8 ​μL of loading buffer (60% (v/v) glycerol, 1 ​mM EDTA, 0.004% (w/v) bromophenol blue, and 0.004% (w/v) xylene cyanol). Gel electrophoresis was performed at room temperature in Tris-borate EDTA buffer (45 ​mM Tris-borate, 1 ​mM EDTA, pH 8.0) on a 2% (w/v) agarose gel at 100 ​V for 30 ​min. The gel was stained with Coomassie Brilliant Blue G250 (Tokyo Chemical Industry Co., Ltd., Tokyo, Japan). The protein bands were visualized using an Amersham Typhoon scanner (FLA-9000, Fujifilm, Tokyo, Japan).

### 2.9 Interaction assay by ultrafiltration

Insulin (0.145 ​mg/mL) or lysozyme (0.5 ​mg/mL) and polymers (NH_2_-PRX, NH_2_-DEX, PEG–NH_2_–PRX, and PEG–NH_2_–DEX) were dissolved in phosphate-buffered saline (PBS) and placed in an Amicon® Ultra cartridge (UFC5050BK, MWCO 50 ​kDa, Merck KGaA, Darmstadt, Germany). The sample was centrifuged at 14,000 ​× ​*g* for 15 ​min, and the absorbance of the filtrate at 220 ​nm (insulin) or 280 ​nm (lysozyme) was measured using a spectrophotometer (NP80, Implen, CA, USA).

### 2.10 Hyaluronan-degrading activity

Hyaluronidase (0.02 ​mg/mL) or covalently PEGylated hyaluronidase (MW of PEG 20 ​kDa) was dissolved in water in the absence and presence of PEG–NH_2_–PRX (0.5 ​mg/mL) and incubated for 10 ​min at 37 ​°C. After adding hyaluronic acid aqueous solution (0.3 ​mg/mL), the sample was incubated for 45 ​min at 37 ​°C. The sample solution (0.5 ​mL) was added to 2.5 ​mL of albumin aqueous solution (1.0 ​mg/mL), and then incubated for 10 ​min at room temperature. The absorbance of the sample at 600 ​nm was measured using a spectrophotometer. Hyaluronan-degrading activity was represented as a relative value for hyaluronidase alone.

### 2.11 Shaking stability

IgG (7.0 ​mg/mL) or panitumumab (5.0 ​mg/mL) was dissolved in PBS or aqueous solution containing 5.824 ​mg/mL NaCl and 6.8 ​mg/mL CH_3_COONa (solvent A) in the absence and presence of 5.0 ​mg/mL (IgG) or 3.57 ​mg/mL (panitumumab) of additives. After shaking at 500 ​rpm and room temperature for 7 days, the sample was diluted with 0.9 ​mL of PBS or solvent A. After centrifugation (12,000 ​rpm, 10 ​min), the absorbance of the supernatant at 280 ​nm was measured using a spectrophotometer. Moreover, to evaluate the stability of highly concentrated IgG, IgG (100 ​mg/mL) was dissolved in PBS in the absence and presence of PEG–NH_2_–PRX (7.1 ​mg/mL) or PEG–NH_2_–DEX (7.1 ​mg/mL). After shaking at 500 ​rpm and room temperature for 7 days, transmittance at 600 ​nm was measured using a microplate spectrophotometer (Epoch, BioTek Japan, Tokyo, Japan).

### 2.12 In vivo hypoglycemic effect

All animal procedures were carried out in accordance with the approved guidelines and with the approval of the Ethics Committee for Animal Care and Use of Kumamoto University (Approval ID: A 2019–077). Insulin (0.145 ​mg/mL) was dissolved in PBS in the absence and presence of PEG–NH_2_–PRX (1.0 ​mg/mL) or PEG–NH_2_–DEX (1.0 ​mg/mL). The samples (insulin 2 U/kg) were subcutaneously injected into male Wistar rats (200–250 ​g) or GK/Slc rats (160–220 ​g), and at appropriate intervals, blood samples were collected from the jugular vein. The serum glucose level of rats was determined by the mutarotase-glucose oxidase method using the Glucose–CII–Test from Wako (Fujifilm Wako Pure Chemical Corporation, Osaka, Japan).

### 2.13 Safety profiles

PEG–NH_2_–PRX (1.0 ​mg/mL) was dissolved in PBS and subcutaneously injected (0.1 ​mL) into male Wistar rats (200–250 ​g) once a day (4 times in total). The body weight of the rats was measured once a day. Twenty-four hours after the final injection, blood samples, and organs (heart, lung, liver, spleen, and kidney) were collected, and the weight of the organs was measured. The blood chemistry values were measured using a clinical chemistry analyzer (Dri-chem 7000V, Fujifilm Corporation, Tokyo, Japan) after injecting the samples.

### 2.14 Data analysis

The quantitative data were expressed as the mean ​± ​standard error of the mean (S.E.), and statistical comparisons were made using Scheffe's test. A *p*-value < 0.05 was considered statistically significant.

## 3 Results and discussion

**Fig. 1:**
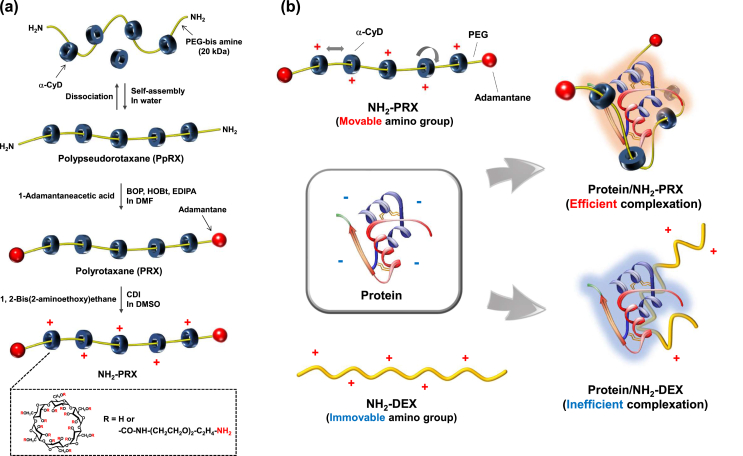
(a) Preparation pathway of NH_2_-PRX and (b) proposed scheme for protein complexation with NH_2_-PRX or NH_2_-DEX.

To prepare a backbone polymer of PEG–NH_2_–PRX, *i.e*. NH_2_-PRX, PRX was first prepared by mixing α-CyD and PEG-bis amine in water and capping with 1-adamantaneacetic acid (yield: 6.5 ​g) (Fig. 1a, S2) [25]. Herein, relatively high-molecular weight PEG (MW 20 ​kDa) was employed as the axile molecule because it allows lower CyD coverage of PRX, and is desirable for moving of CyD molecule in PRX [27]. Next, to obtain NH_2_-PRX, PRX was activated by CDI and reacted with 1,2-bis(2-aminoethoxy)ethane (Fig. 1a) [26]. The ^1^H NMR spectrum of NH_2_-PRX showed peaks derived from α-CyD, PEG, and 1,2-bis(2-aminoethoxy)ethane, indicating the successful preparation of NH_2_-PRX (Fig. S3). The number of α-CyD calculated from the peak area of the anomeric proton of α-CyD and the ethylene proton of PEG was 59.2 with 26% of coverage (Table 1). The degree of substitution (DS) of the amino group per PRX molecule was 208 (3.5 *per* α-CyD). Moreover, NH_2_-DEX with 178 amino groups in one molecule was also prepared from dextran (MW 70 ​kDa) according to the methods reported previously (Fig. S4, S5, Table 1) [26].

**Table 1:** Characterization of NH_2_-PRX and NH_2_-DEX.

Compound	Number of α-CyD	Coverage (%)^a^	Number of NH_2_	MW (kDa)
NH_2_-PRX	59.2	26.0	208	114
NH_2_-DEX	–	–	178	101

a Coverage was determined as percentage of the number of α-CyD against the maximum number of α-CyD threaded onto 20 ​kDa PEG (*ca*. 227).

**Fig. 2:**
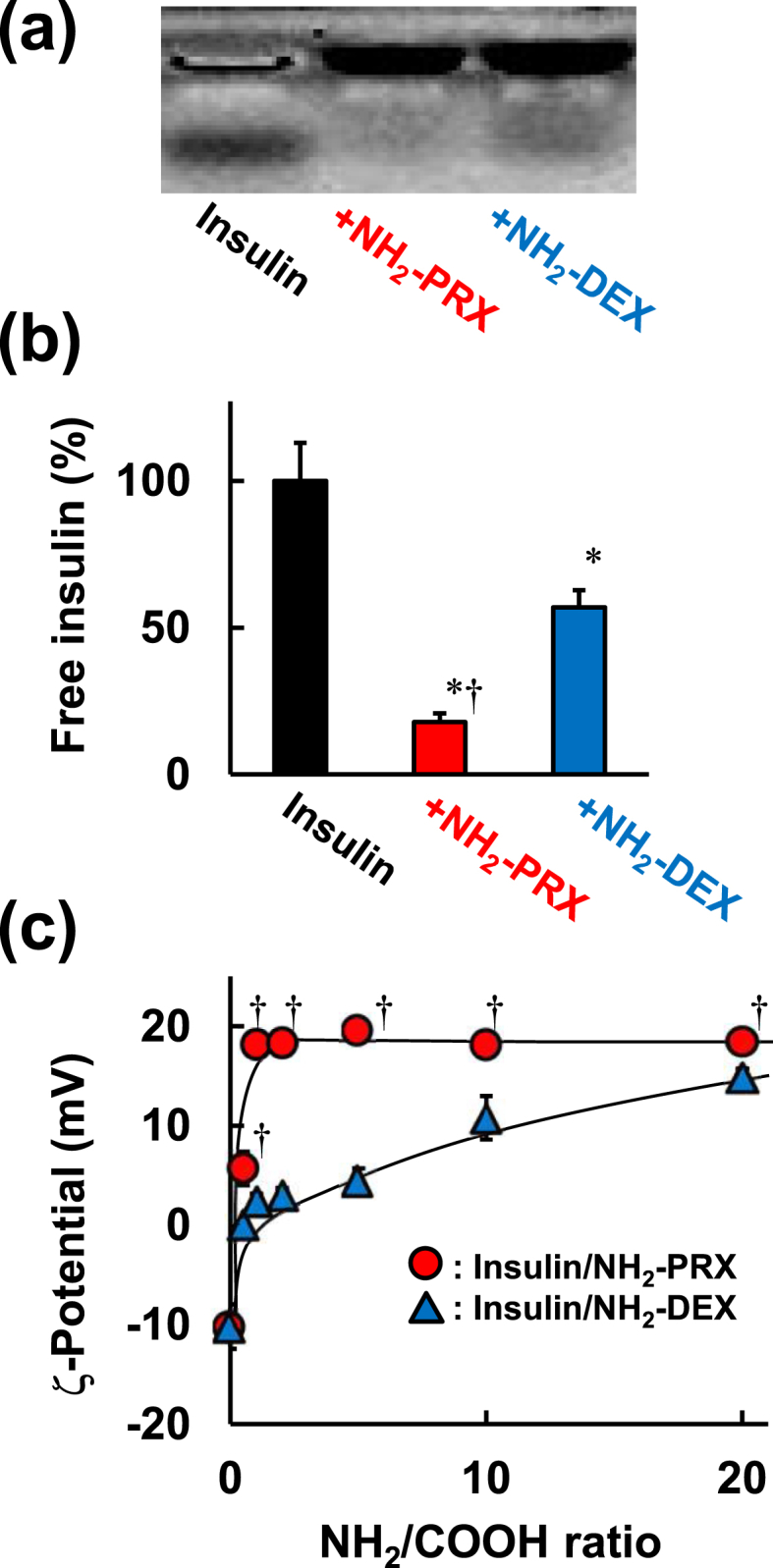
Interaction between protein and NH_2_-PRX or NH_2_-DEX. (a) Agarose gel electrophoretic permeation, (b) permeation through an ultrafiltration membrane and (c) ζ-potential of insulin in the absence and presence of NH_2_-PRX or NH_2_-DEX. ∗*p* ​< ​0.05 *vs.* insulin. †*p* ​< ​0.05 *vs.* insulin/NH_2_-DEX. n ​= ​3–4.

To examine the interaction between insulin and NH_2_-PRX, gel electrophoresis was performed (Fig. 2a). The band derived from free insulin in insulin/NH_2_-PRX was lighter than that in insulin/NH_2_-DEX, indicating strong interaction between insulin and NH_2_-PRX. The complexation of insulin/NH_2_-PRX was also evaluated by ultrafiltration (Fig. 2b), and the amount of free insulin in the filtrate of insulin/NH_2_-PRX was smaller than that of insulin/NH_2_-DEX. Moreover, to ensure the efficient complexation between insulin and NH_2_-PRX, the ζ-potential of insulin was measured. The ζ-potential of insulin was markedly changed to positive by adding NH_2_-PRX at a lower NH_2_/COOH ratio of polymer/insulin compared to NH_2_-DEX (Fig. 2c). Surprisingly, ζ-potential of insulin/NH_2_-PRX reach a plateau at NH_2_/COOH ratio 1, although insulin/NH_2_-DEX did at NH_2_/COOH ratio >20. This strongly suggests that insulin/NH_2_-PRX forms a complex more efficiently than insulin/NH_2_-DEX. Importantly, the number of amino group and total molecular weight were almost same between NH_2_-PRX and NH_2_-DEX (Table 1), suggesting that the different complexation ability between NH_2_-PRX and NH_2_-DEX is not due to difference of their chemical characteristics. As described above, amino groups in NH_2_-PRX are movable onto the axile molecule. Thus, NH_2_-PRX could provide amino groups to insulin on demand, whereas the lower mobility of amino groups in NH_2_-DEX resulted in structural mismatch between insulin and NH_2_-DEX (Fig. 1b). Most recently, sliding motion of CyD in PRX was firstly demonstrated by means of quasi-elastic neutron scattering [18]. As future efforts, we should demonstrate movable properties of CyD and amino groups in NH_2_-PRX directly.

**Fig. 3:**
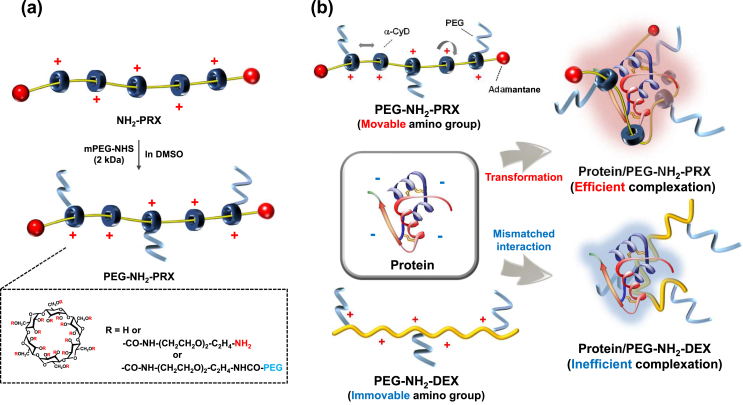
(a) Preparation pathway of PEG–NH_2_–PRX and (b) proposed scheme for protein complexation with PEG–NH_2_–PRX or PEG–NH_2_–DEX.

Next, to fabricate the transformable mixing-type PEGylation material, *i.e*. PEG–NH_2_–PRX, PEG was partially grafted with the amino groups of NH_2_-PRX (yield: 178.1 ​mg) (Fig. 3a). Here, relatively low-molecular weight PEG (MW: 2 ​kDa) was employed because high-molecular weight PEG could have attenuated the interaction between PEG–NH_2_–PRX and proteins because of steric hindrance. In contrast, graft of a number of 2 ​kDa PEG may be sufficient for avoiding glomerular filtration without high steric hindrance. Based on the ^1^H NMR spectrum (Fig. S6), the numbers of PEG chains and amino groups in one PRX were calculated as 23 and 185, respectively (Table 2). Hence, the total average MW of PEG–NH_2_–PRX was 160 ​kDa, which is sufficient for avoiding glomerular filtration. As a control possessing low mobility of amino groups (Fig. 3b), PEG–NH_2_–DEX was prepared using similar methods (yield: 97.1 ​mg) (Fig. S7), and characterized by ^1^H NMR (Fig. S8). The numbers of PEG chains and amino groups in one DEX were calculated as 20 and 166, respectively, with 142 ​kDa total average MW (Table 2).

**Table 2:** Characterization of PEG–NH_2_–PRX and PEG–NH_2_–DEX.

Compound	Number of PEG	Number of NH_2_	MW (kDa)
PEG–NH_2_–PRX	23	185	160
PEG–NH_2_–DEX	20	166	142

**Fig. 4:**
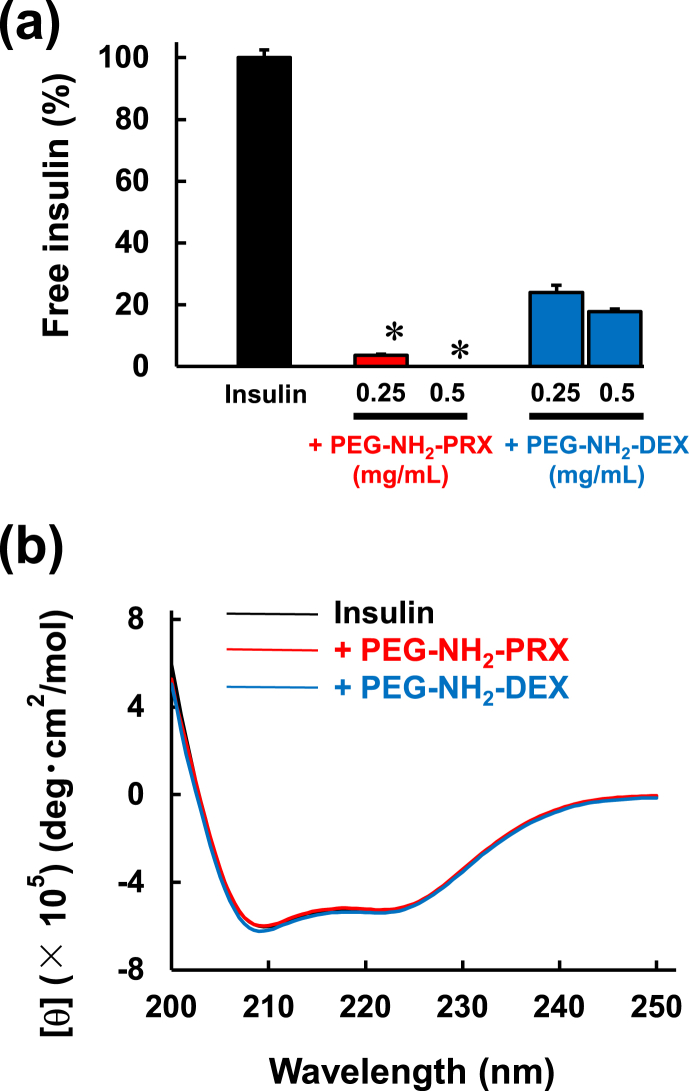
(a) Permeation through the ultrafiltration membrane and (b) CD spectrum of insulin in the absence and presence of PEG–NH_2_–PRX or PEG–NH_2_–DEX. ∗*p* ​< ​0.05 *vs*. insulin/PEG–NH_2_–DEX. n ​= ​3–4.

The interaction between insulin and PEG–NH_2_–PRX was evaluated by ultrafiltration (Fig. 4a). The amount of free insulin was reduced by PEG–NH_2_–PRX addition, and was remarkable compared to PEG–NH_2_–DEX. Thus, as expected, PEG–NH_2_–PRX interacted with insulin more efficiently than PEG–NH_2_–DEX (Fig. 3b). Nelson et al. reported that lactose-appended PpRX interacts more strongly with galectin-1 than lactose-appended α-CyD [28]. Ooya et al. also reported that the interaction of maltose-appended PRX with concanavalin A is 367-times greater than that of maltose-appended α-CyD [21,22]. Thus, the efficiency of interaction with the target molecules results from the PRX-structure. Hereafter, we shall compare the interaction of proteins/PEG–NH_2_–PRX and proteins/PEG–NH_2_–α-CyD to demonstrate the importance of PRX-structure.

As shown in Fig. S9, PEG–NH_2_–PRX negligibly formed the complex with basic protein, lysozyme. These results strongly suggest that efficient complexation takes place between acidic proteins and PEG–NH_2_–PRX, probably due to the interaction between acidic amino acid residues of proteins and amino groups of PEG–NH_2_–PRX. In future, we develop acidic PEGylation materials for basic proteins using PRX with anionic groups and PEG chains.

To examine the conformation of insulin in the complexes, CD spectra were obtained (Fig. 4b). The CD spectrum of insulin was negligibly changed by the addition of PEG–NH_2_–PRX or PEG–NH_2_–DEX, indicating that insulin in these complexes retained its conformation. Moreover, PEG–NH_2_–PRX/hyaluronidase, another model protein, exhibited *ca*. 95% hyaluronan-degrading activity, compared with that of hyaluronidase alone (Fig. S10), while the activity of covalently PEGylated hyaluronidase with PEG 20 ​kDa was 27% (Fig. S11). The results suggested that PEG–NH_2_–PRX could be used for proteins other than insulin, and that it negligibly changed the conformation and activity of the protein drugs.

**Fig. 5:**
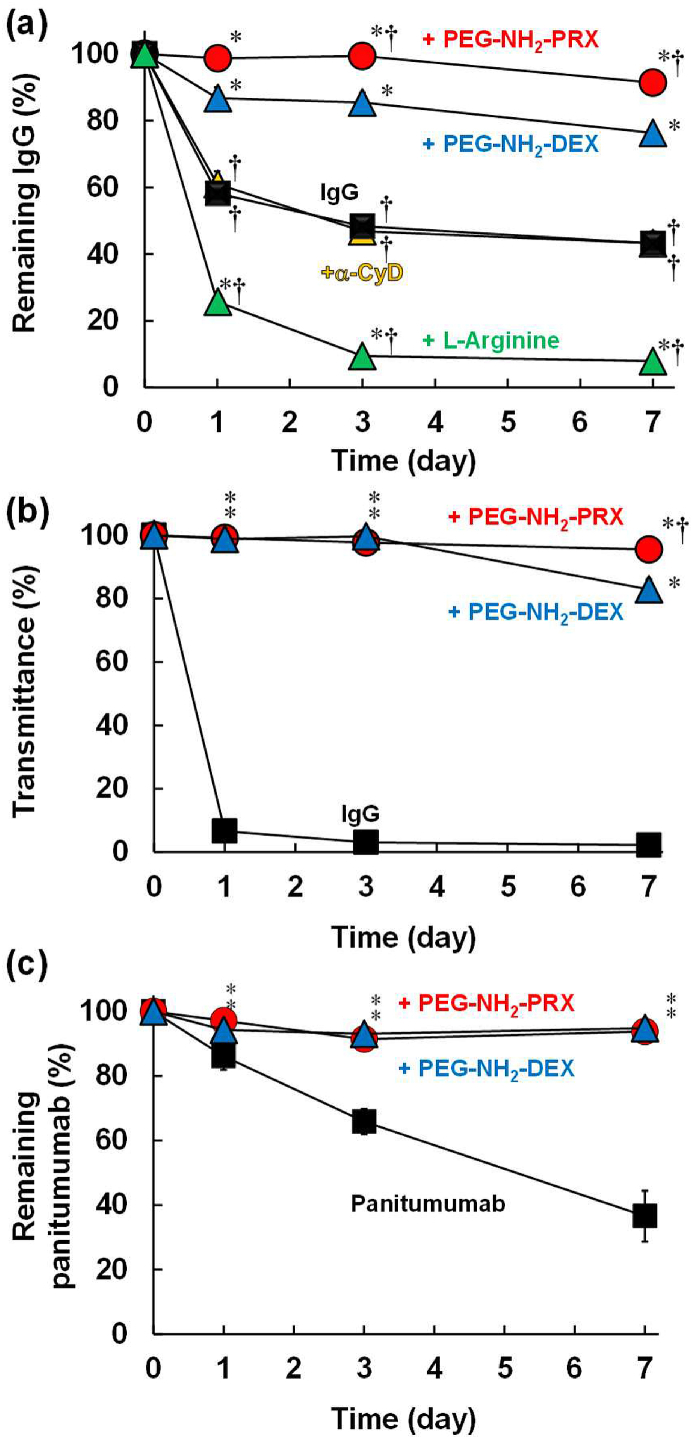
Effects of PEG–NH_2_–PRX or PEG–NH_2_–DEX on the shaking stability of (a) IgG (7 ​mg/mL), (b) IgG (100 ​mg/mL), and (c) panitumumab (5 ​mg/mL). ∗*p* ​< ​0.05 *vs.* IgG or panitumumab. †*p* ​< ​0.05 *vs.* IgG/PEG–NH_2_–DEX. n ​= ​3–6.

In recent years, antibody drugs have been developed extensively; however, they often form aggregates, resulting in poor product quality. Thus, the effects of PEG–NH_2_–PRX on antibody stability against shaking stress were evaluated. As shown in Fig. 5a, *ca*. 57% of IgG formed aggregates after shaking (500 ​rpm for 7 days) at a low concentration (7 ​mg/mL), and α-CyD showed the negligible stabilizing effects. Contrary to expectations, L-arginine, a general protein stabilizer, enhanced aggregates of IgG under the present experimental conditions. The reason behind this observation is unclear; however, reportedly, arginine works as both a protein stabilizer and destabilizer in liquid formulations [29]. On the other hand, both PEG–NH_2_–PRX and PEG–NH_2_–DEX dramatically inhibited IgG aggregation; in particular, the stabilizing effects of PEG–NH_2_–PRX were significantly higher than those of PEG–NH_2_–DEX. Thus, PEG–NH_2_–PRX exhibits strong stabilizing effects on antibodies. Meanwhile, though highly concentrated antibody drugs are often developed in the clinical setting, they form aggregates easily. Therefore, the stabilizing effects of PEG–NH_2_–PRX on highly concentrated IgG formulations were examined. As shown in Fig. 5b, PEG–NH_2_–PRX markedly inhibited IgG aggregation at 100 ​mg/mL of IgG, indicating its strong stabilizing effects. Moreover, PEG–NH_2_–PRX also showed marked stabilizing effects with a commercially available antibody drug, panitumumab (Fig. 5c). In the case of panitumumab, no statistically significant difference was observed between the stabilizing effects of PEG–NH_2_–PRX and PEG–NH_2_–DEX because of the high stability of panitumumab in the PEG–NH_2_–DEX complex. Hereafter, we shall compare the stability at severer conditions. Anyhow, these results indicate the potential of PEG–NH_2_–PRX as a stabilizing agent for antibody drugs.

**Fig. 6:**
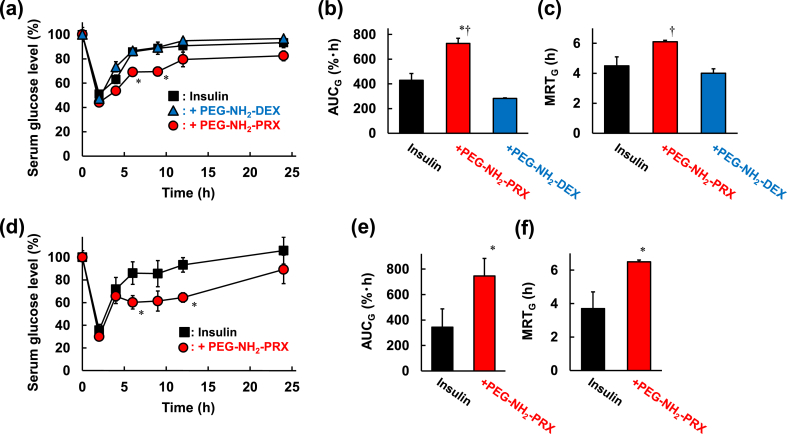
(a, d) Serum glucose levels, (b, e) AUC_G_ and (c, f) MRT_G_ after subcutaneous administration of insulin, insulin/PEG–NH_2_–PRX, or insulin/PEG–NH_2_–DEX to (a–c) healthy rats and (d–f) GK/Slc diabetes model rats. ∗*p* ​< ​0.05 *vs.* insulin. †*p* ​< ​0.05 *vs.* insulin/PEG–NH_2_–DEX. n ​= ​4–6.

To evaluate the sustained effects of PEG–NH_2_–PRX on the *in vivo* bioactivity of protein drugs, the hypoglycemic effect of insulin/PEG–NH_2_–PRX was evaluated in healthy rats (Fig. 6a). Low-serum glucose levels were prolonged after subcutaneous administration of insulin/PEG–NH_2_–PRX, compared with administration of both insulin alone and insulin/PEG–NH_2_–DEX. Based on the serum glucose levels, the area upper the time-curve for serum glucose level up to 24 ​h post-administration (AUC_G_) (Fig. 6b) and the mean residence time of serum glucose levels (MRT_G_) (Fig. 6c) were determined as indexes for the magnitude and duration of *in vivo* insulin bioactivity, respectively. Both the AUC_G_ and MRT_G_ of insulin/PEG–NH_2_–PRX were higher than those of both insulin alone and of insulin/PEG–NH_2_–DEX, suggesting that PEG–NH_2_–PRX sustained the hypoglycemic effect of insulin without loss of activity. Importantly, we previously reported that the covalently PEGylated insulin with PEG 20 ​kDa showed only 10% hypoglycemic effect versus insulin alone in the almost same experimental conditions [11]. Therefore, PEG–NH_2_–PRX could be a promising PEGylation method beyond the conventional method, namely covalent PEGylation.

PEGylation forms a hydration layer on the surface of proteins, leading to low affinity to a target molecule and decrease of the activity. Thus, to exhibit the activity, reversibly PEGylated proteins should dissociate. In fact, we previously demonstrated that the activity of reversibly PEGylated protein is increased by dilution probably due to the acceleration of the dissociation [10]. Therefore, insulin/PEG–NH_2_–PRX probably exhibits the hypoglycemic effects through dilution and subsequent dissociation in the subcutaneous tissues or blood. In addition, molecular dynamics and structure of PRX get altered under various conditions [18,30]; therefore, conformation of PEG–NH_2_–PRX might be changed by dilution, which may lead to the acceleration of dissociation. In the future, the changes in the conformation of PEG–NH_2_–PRX as a result of dilution should be studied. Moreover, competitive interaction of insulin/endogenous albumin with PEG–NH_2_–PRX or insulin receptor/PEG–NH_2_–PRX with insulin may accelerate the dissociation of insulin/PEG–NH_2_–PRX. Therefore, we evaluated the interaction between human serum albumin (HSA) and PEG–NH_2_–PRX (Fig. S12). The negative charge of free HSA (−12.7 ​mV) was increased by the addition of PEG–NH_2_–PRX in a concentration-dependent manner, indicating the interaction of PEG–NH_2_–PRX with albumin. Thus, the competitive interaction with albumin could be one of the factors responsible for the dissociation of insulin/PEG–NH_2_–PRX, and the lag time until insulin in the PEG–NH_2_–PRX complex is replaced by albumin could lead to the duration of the hypoglycemic effect.

Next, to determine the utility of insulin/PEG–NH_2_–PRX as a long-acting insulin product, its hypoglycemic effect was evaluated in the GK/Slc rat model of type 2 diabetes. The hypoglycemic effect of insulin/PEG–NH_2_–PRX was sustained, compared with that of insulin alone (Fig. 6d). Moreover, the AUC_G_ (Fig. 6e) and MRT_G_ (Fig. 6f) were significantly higher than those of insulin alone, indicating the potential of insulin/PEG–NH_2_–PRX as a long-acting insulin product.

We previously developed a monovalent-type reversible PEGylated insulin through host-guest interactions between β-CyD and adamantane [11]. This also showed prolonged hypoglycemic effect *in vivo*; however chemical modification of adamantane was required. PEG–NH_2_–PRX allows reversible PEGylation by only mixing with proteins. Therefore, PEG–NH_2_–PRX has the great potentials as the advanced materials to improve the pharmaceutical properties of protein drugs.

**Fig. 7:**
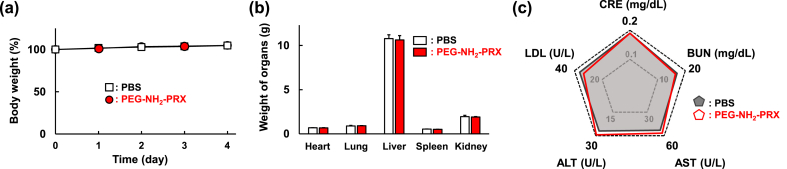
(a) Body weight, (b) weight of organs and (c) blood chemistry values after 4 subcutaneous doses of PBS or PEG–NH_2_–PRX administered to healthy rats. CRE, creatinine; BUN, blood urea nitrogen; AST, aspartate aminotransferase; ALT, alanine aminotransferase; LDL, lactate dehydrogenase. n ​= ​6.

Finally, to estimate the *in vivo* safety profiles of PEG–NH_2_–PRX, body weight (Fig. 7a), organ weight (Fig. 7b), and blood chemistry values (Fig. 7c) were measured after administering four subcutaneous doses of PEG–NH_2_–PRX to healthy rats. Negligible changes in body weight, organ weight, and blood chemistry values were observed, compared with those in the administration of PBS, suggesting the safety of PEG–NH_2_–PRX.

## 4 Conclusions

In conclusion, we developed a transformable mixing-type material, PEG–NH_2_–PRX, for the reversible PEGylation of protein drugs. PEG–NH_2_–PRX efficiently formed complexes with proteins and markedly improved the stability and duration of *in vivo* bioactivity of proteins, compared with PEG–NH_2_–DEX. These findings suggest that the supramolecular material, PEG–NH_2_–PRX, is a promising PEGylation material for protein drugs, compared with macromolecular materials.

## Credit author statement

Kosei Utatsu, Tetsuya Kogo: Data curation, Formal analysis, Investigation, Methodology, Validation, Writing – review & editing. Toru Taharabaru: Data curation, Formal analysis, Investigation, Methodology, Validation. Risako Onodera: Investigation, Methodology, Writing – review & editing. Keiichi Motoyama: Investigation, Methodology, Supervision, Writing – review & editing. Taishi Higashi: Conceptualization, Funding acquisition, Investigation, Methodology, Supervision, Writing – original draft, Writing – review & editing.

## Declaration of competing interest

The authors declare that they have no known competing financial interests or personal relationships that could have appeared to influence the work reported in this paper.
