# Supplementary material for: Supramolecular polymer-based transformable material for reversible PEGylation of protein drugs
Source: Mater Today Bio. 2021 Nov 16;12:100160. doi: 10.1016/j.mtbio.2021.100160 (PMC8605344; doi:10.1016/j.mtbio.2021.100160)
Supplement: Multimedia component 1 [file mmc1.pdf]

Supplementary Information

Supramolecular polymer-based transformable material for reversible PEGylation of protein drugs

Kosei Utatsu, Tetsuya Kogo, Toru Taharabaru, Risako Onodera, Keiichi Motoyama, Taishi Higashi*

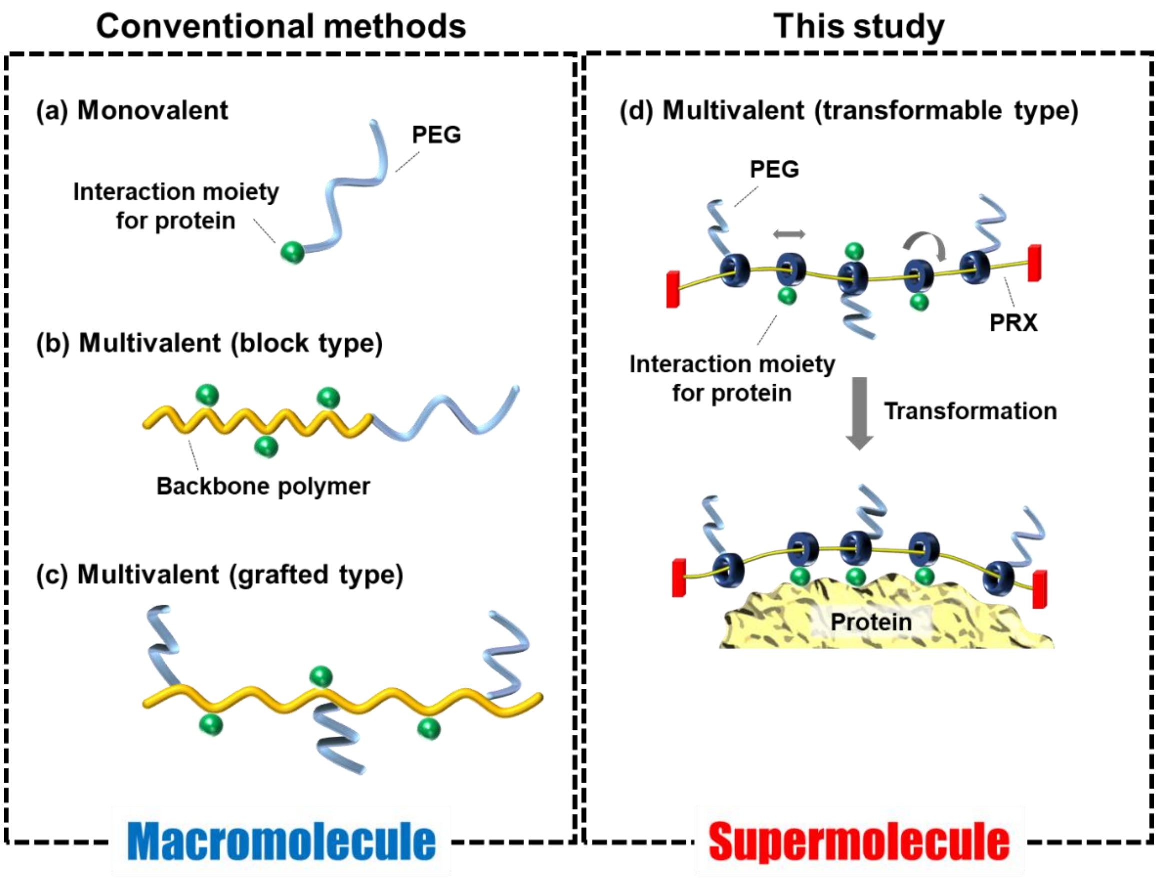

Fig. S1. Classification of mixing-type PEGylation materials.

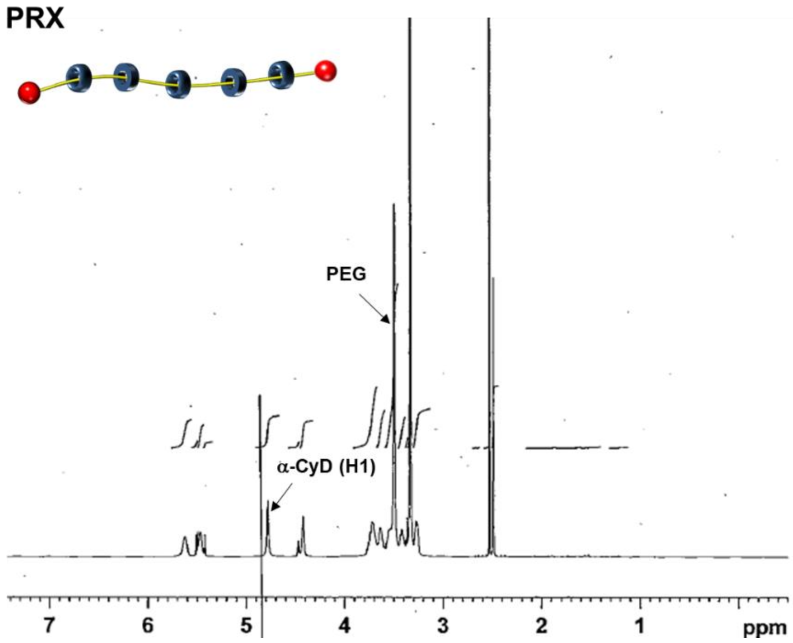

Fig. S2. ^{1}H-NMR spectrum of PRX in DMSO-d_6.

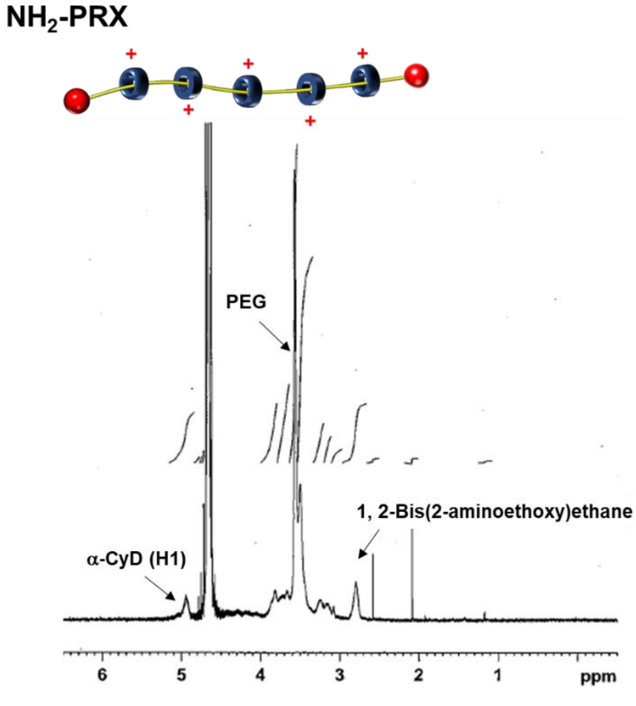

Fig. S3. ^{1}H-NMR spectrum of NH_2-PRX in D_2O.

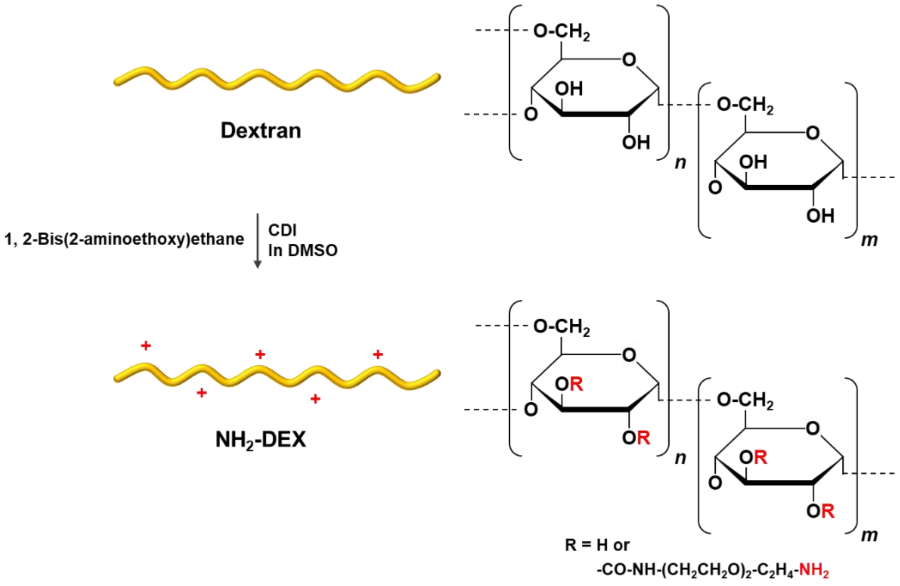

Fig. S4. Preparation pathway of NH_2-DEX.

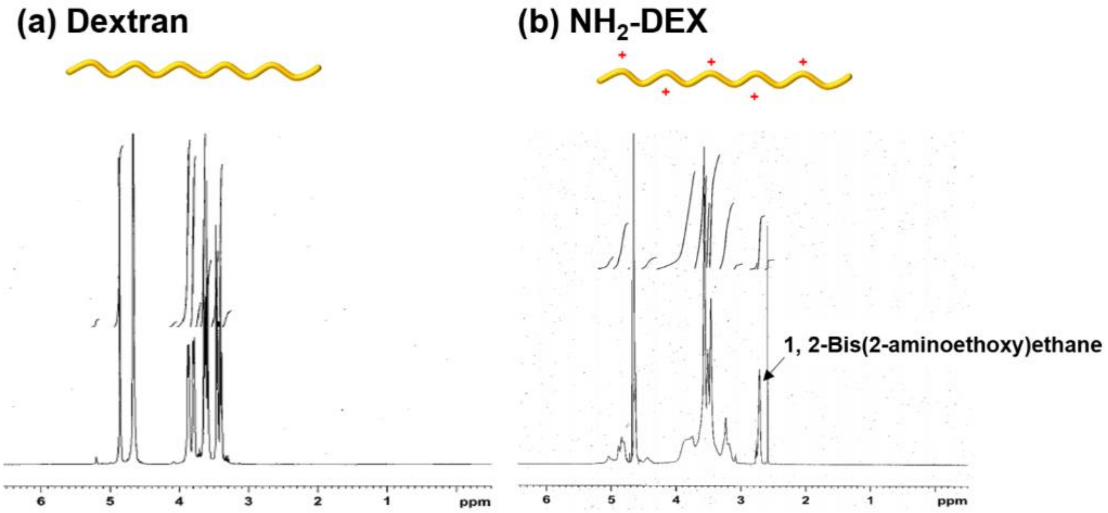

Fig. S5. ^{1}H-NMR spectra of (a) dextran and (b) NH_2-DEX in D_2O.

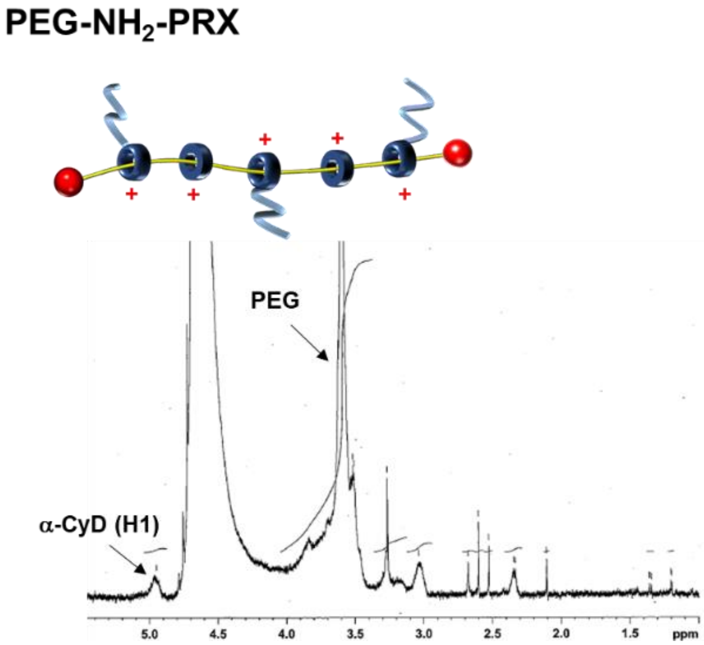

Fig. S6. ^{1}H-NMR spectrum of PEG-NH$_2$-PRX in D$_2$O.

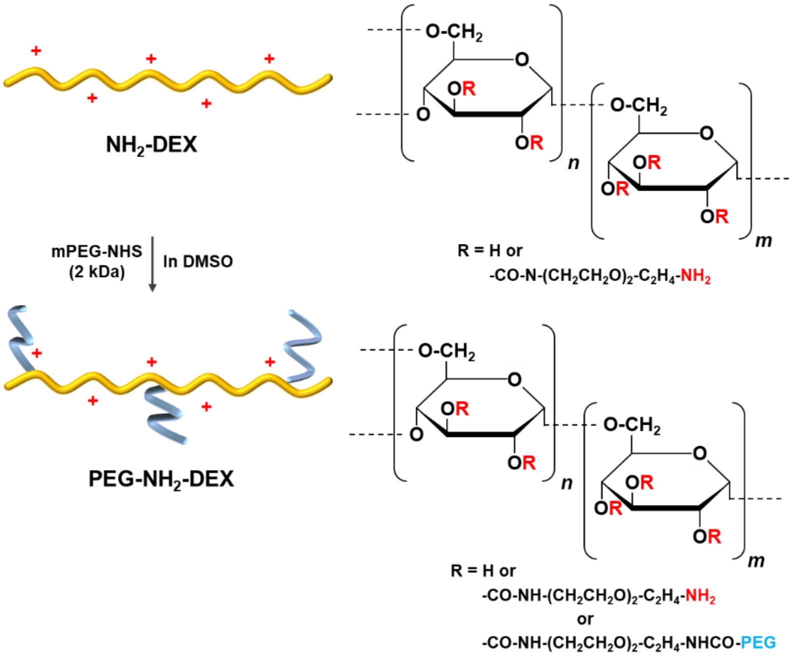

Fig. S7. Preparation pathway of PEG-NH_2-DEX.

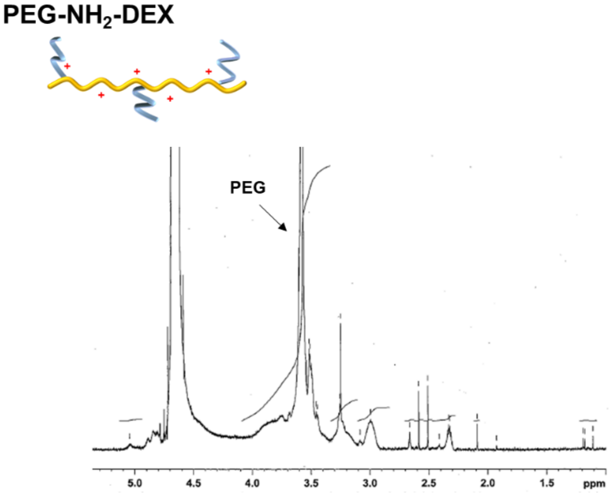

Fig. S8. ^{1}H-NMR spectrum of PEG-NH_2-DEX in D_2O.

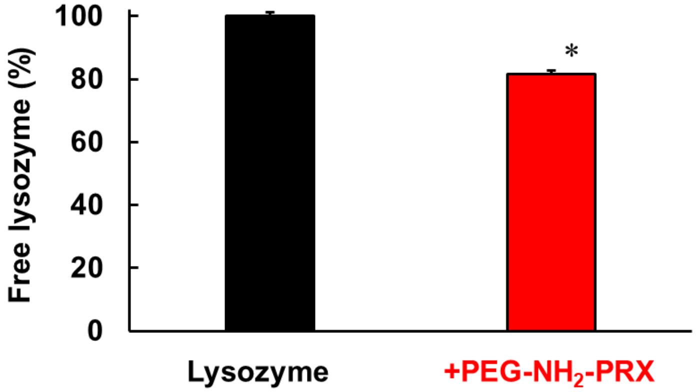

Fig. S9. Permeation of lysozyme through an ultrafiltration membrane in the absence and presence of PEG-NH_2-PRX. *p < 0.05 *vs.* lysozyme. n=3.

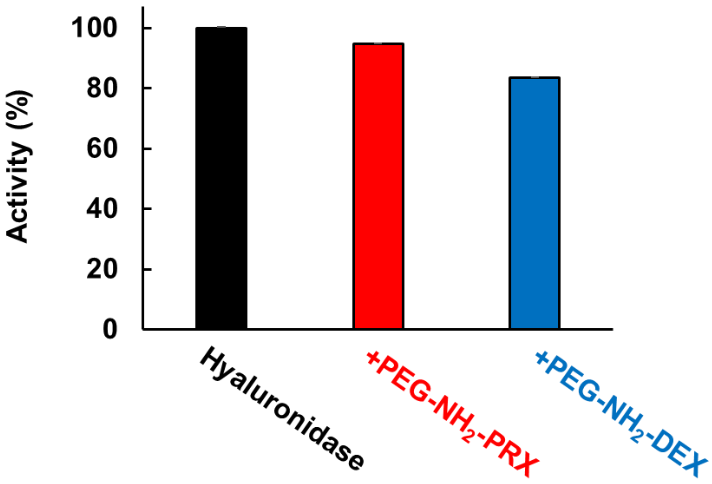

Fig. S10. *In vitro* hyaluronan-degrading activity of hyaluronidase, hyaluronidase/PEG-NH_2-PRX, and hyaluronidase/PEG-NH_2-DEX. n=3.

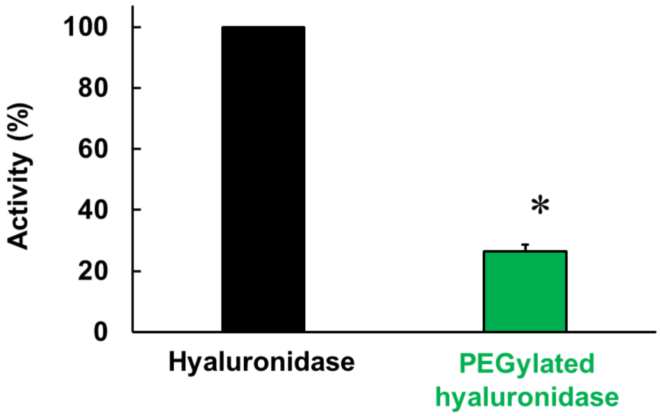

Fig. S11. *In vitro* hyaluronan-degrading activity of hyaluronidase and covalently PEGylated hyaluronidase. *$p < 0.05$ *vs.* hyaluronidase. n=3.

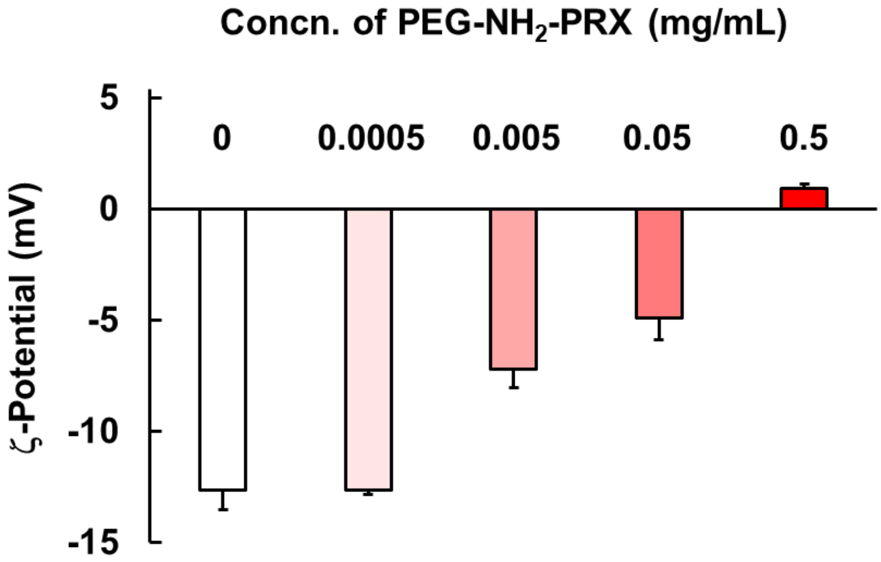

Fig. S12. ζ-Potentials of the mixtures of human serum albumin (HSA) and PEG-NH_2-PRX. n=4. HSA (0.5 mg/mL) and PEG-NH_2-PRX (0.0005-0.5 mg/mL) were dissolved in HBSS (pH 7.4), and incubated for 30 min at room temperature. The ζ-potential values were determined using a Zetasizer Nano ZS apparatus.
